# Distal revascularization and interval ligation for femoral arteriovenous graft steal syndrome

**DOI:** 10.1016/j.jvscit.2026.102255

**Published:** 2026-04-11

**Authors:** Francesca Osborn, Max Murray-Ramcharan, Pooja Balar, Mina Iskaros, S. Christopher Frontario, Thomas Bernik

**Affiliations:** a Department of Environmental and Biologic Sciences, Rutger’s University, New Brunswick, NJ; b Department of Vascular Surgery, Englewood Health, Englewood, NJ; c Drexel University College of Medicine, Philadelphia, PA

**Keywords:** Distal revascularization-interval ligation, DRIL, Hemodialysis, Femoral arteriovenous access, Femoral arteriovenous graft, AVG, Steal syndrome, Limb ischemia

## Abstract

Patients with end-stage renal disease and exhausted upper extremity options may require lower extremity arteriovenous grafts (AVGs) for hemodialysis access. We present a 62-year-old woman with a right superficial femoral artery-femoral vein looped AVG who developed limb-threatening ischemia secondary to steal syndrome. A distal revascularization and interval ligation procedure, using an external iliac-to-distal superficial femoral artery bypass with interval ligation, successfully restored perfusion while preserving access. The patient's ischemic symptoms resolved, and both AVG and bypass remained patent at >2 years, demonstrating distal revascularization and interval ligation as an effective limb-salvaging option.

In the United States, >400,000 individuals are on hemodialysis (HD).^1^ According to the National Kidney Foundation Kidney Disease Outcomes Quality Initiative and the Fistula First Breakthrough Initiative, the preferred access for HD is an upper extremity autogenous arteriovenous fistula (AVF), with an arteriovenous graft (AVG) reserved for patients lacking adequate venous anatomy. Lower extremity AVFs or AVGs are typically considered only after all upper extremity options have been exhausted.^2^, ^3^, ^4^, ^5^

Although not first line, lower extremity access can offer acceptable patency rates, often superior to catheter-based alternatives.^5^^,^^6^ However, these procedures may carry an increased risk for complications such as limb ischemia, infection, and steal syndrome.^7^

Dialysis-associated steal syndrome (DASS) is an uncommon but potentially devastating complication of AVFs or AVGs, with an incidence of 1% to 8%.^8^^,^^9^ Presentation ranges from mild HD-related symptoms to severe ischemia and tissue loss, including digital gangrene. DASS is more frequently associated with AVGs than AVFs^10^ and occurs in ≤5% of upper extremity accesses in some series.^11^, ^12^, ^13^ Reports of DASS in lower extremity accesses are limited, but small series estimate an incidence of 1% to 7%.^3^^,^^14^

Among surgical management strategies for DASS, distal revascularization and interval ligation (DRIL) is often favored when intervention is warranted.^15^ Originally described for upper extremity DASS, DRIL involves creating a bypass to restore distal perfusion while ligating the native artery distal to the AV anastomosis, thereby preserving access flow.^16^

For patients with femoral AV accesses, DASS is rarely reported, and published examples of successful salvage with DRIL are exceedingly few.^17^^,^^18^ Here, we describe the technical details and outcomes of a DRIL procedure performed to treat steal syndrome in a patient with a femoral AVG. The patient provided written consent for case details and images to be published.

## Case report

This is a 62-year-old woman nonsmoker, with a history of HIV and HIV associated end-stage renal disease on HD for >25 years. She has no history of peripheral artery disease or other significant risk factors, and her vascular access history included multiple bilateral upper extremity fistulas and grafts, each requiring repeat open and endovascular revisions. She subsequently developed superior vena cava syndrome, which was managed with superior vena cava and bilateral brachiocephalic stents, but ultimately this precluded further upper extremity access attempts.

**Fig 1:**
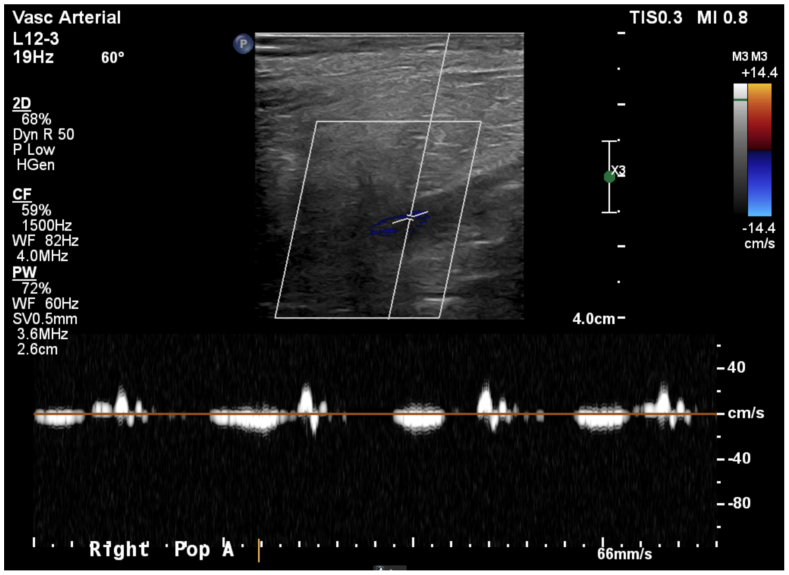
Duplex ultrasound demonstrating presteal physiology in distal superficial femoral artery (SFA) and popliteal artery.

Given her relatively young age and exhausted upper extremity options, a right lower extremity looped AVG was considered. The patient had palpable pulses at all levels in bilateral lower extremities, and an arterial duplex demonstrated no significant atherosclerotic disease. The AVG was fashioned between the proximal superficial femoral artery (SFA) and adjacent femoral vein using a 5-mm Artegraft (LeMaitre Vascular). Distal pulses remained palpable postoperatively. The graft functioned well for >1 year without any presence of claudication, rest pain, or physical examination changes before the patient developed a painful, nonhealing medial ankle wound. At this time, only distal signals were present, and duplex ultrasound examination demonstrated a patent AVG but bidirectional flow in the distal SFA and popliteal artery (Fig 1), consistent with steal physiology. Angiography confirmed a patent graft with sluggish distal flow, patency of the iliac and femoral arteries, and triple vessel runoff to the foot with no evidence of significant atherosclerotic disease. The physical examination along with objective demonstration of steal physiology confirmed the diagnosis, and further testing was not required.

**Fig 2:**
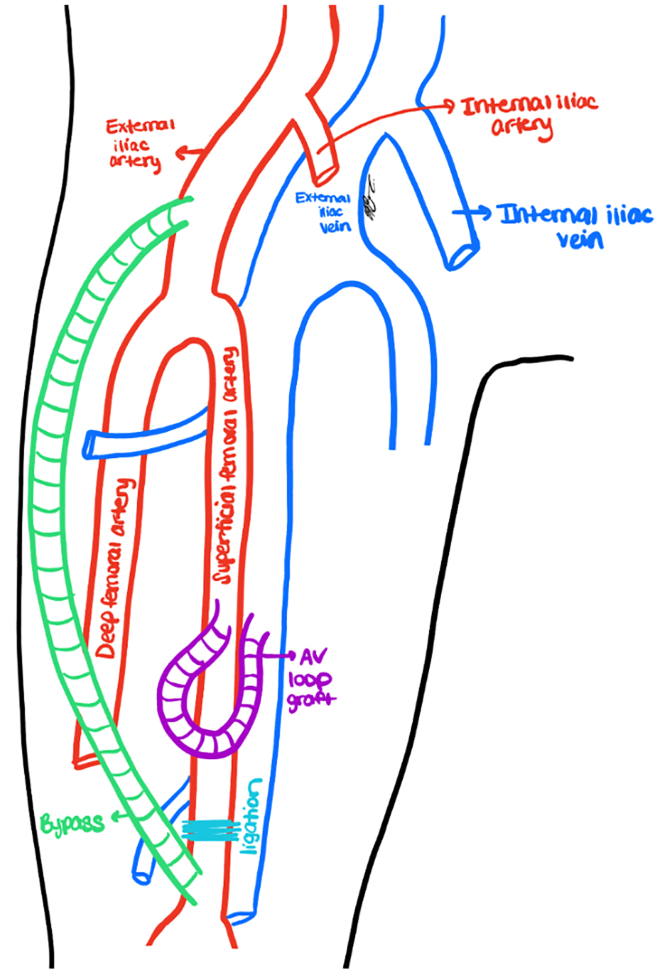
Diagram of preoperative planning for proposed femoral distal revascularization interval ligation, showing an external iliac to superficial femoral artery (SFA) bypass, distal SFA ligation, and existing femoral arteriovenous loop graft. *AV*, arteriovenous.

**Fig 3:**
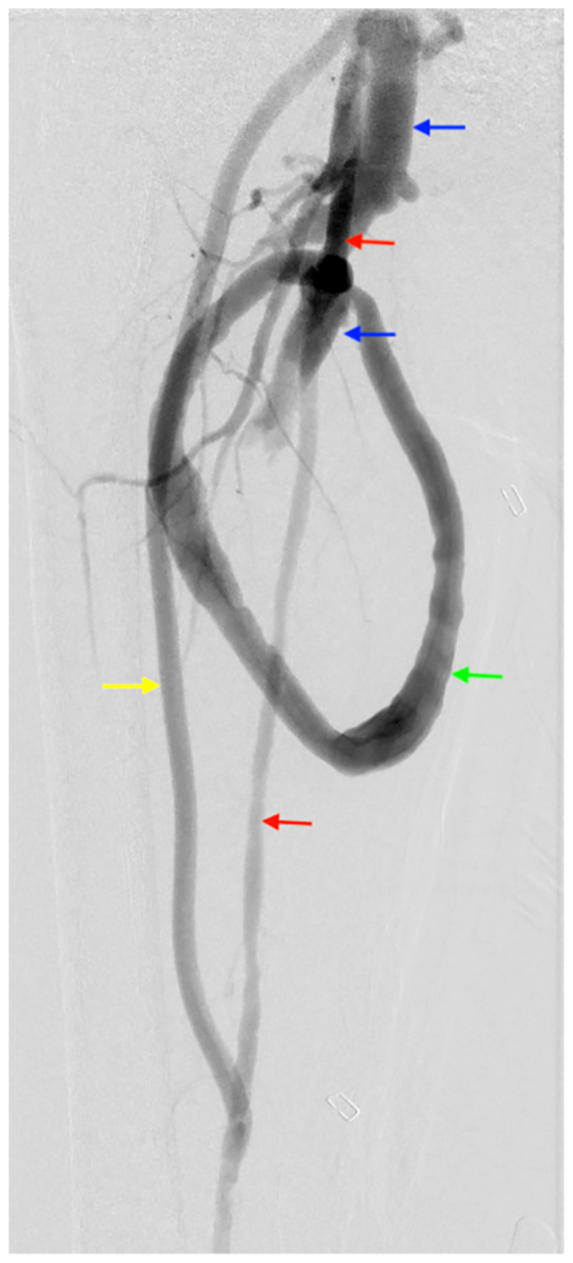
Angiogram of completed external iliac to superficial femoral artery (SFA) bypass (*yellow arrow*) before ligation of SFA (*red arrows*). Note femoral vein (*blue arrows*) and femoral arteriovenous loop graft (*green arrow*).

**Fig 4:**
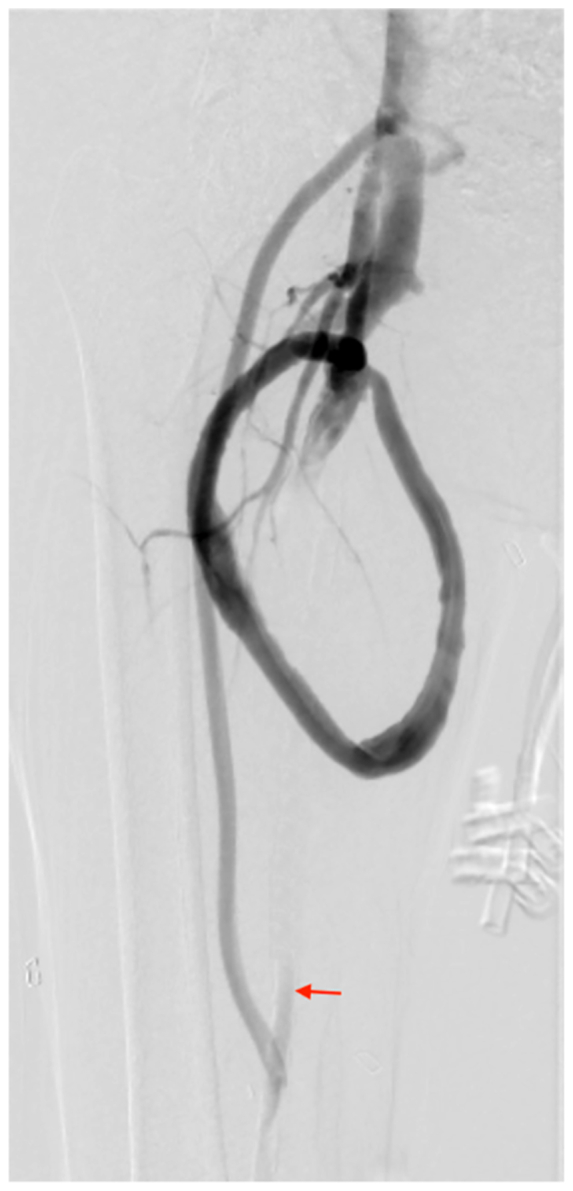
Angiogram of right lower extremity with completed distal revascularization interval ligation anatomy following ligation of superficial femoral artery (SFA) (*red arrow*).

To preserve the functional AVG while addressing limb ischemia, a DRIL procedure was performed, modified for lower extremity anatomy (Fig 2). The right external iliac artery was exposed via a horizontal retroperitoneal incision as the location for the proximal anastomosis to avoid the prior reoperative field at the femoral level, and distal SFA (approximately 20 cm distal to the AVG anastomosis) was similarly exposed for the distal anastomosis. No suitable autogenous venous conduit was available, and a 6-mm ringed polytetrafluoroethylene graft was used for bypass tunneled in a standard subsartorial fashion, restoring antegrade flow to the distal lower extremity. Angiography confirmed bypass patency (Fig 3), after which the SFA was ligated proximal to the distal bypass anastomosis. Completion angiography demonstrated an appropriate DRIL configuration with restored perfusion to the foot and preserved AVG flow (Fig 4).

The postoperative course was unremarkable, the patient was discharged 4 days later, and the AVG remained functional. Because the patient has no history of peripheral artery disease, no antiplatelets or anticoagulation were required, and the patient was seen at standard follow-up intervals with physical examination and duplex surveillance performed at each visit. The patient's ischemic wound healed completely within 3 months, and both the AVG and DRIL bypass remained functional and widely patent at the 3-year follow-up with appropriate waveform patterns and velocities, no additional reinterventions and no recurrence of symptoms.

## Discussion

Patients with end-stage renal disease often present with limited options for upper extremity access owing to recurrent thrombosis, repeated revisions, and central venous stenosis.^19^ When upper extremity options are exhausted, lower extremity access represents a viable, albeit last resort, alternative.^20^ As demonstrated in this case, femoral loop AVGs can provide durable HD access, with acceptable patency and complication rates compared with catheter-based options.^21^, ^22^, ^23^, ^24^, ^25^

AVG flow tends to be more stable as compared with fistulas, and it is essential to rule out intrinsic stenoses, kinks, or other causes of volumetric disturbances when considering DASS.^8^ In this case, authors speculate that, despite a reassuring examination after AVG creation, this patient may have had a subtle, asymptomatic steal that manifested as a wound in the following years. Although upper extremity DASS is well-documented, reports of steal syndrome from femoral AVGs are infrequently described.^26^ Lower extremity steal tends to be particularly severe, with 1% to 7% of patients experiencing ischemic complications that may progress to critical limb ischemia and limb loss.^6^^,^^21^ Extrapolating from the upper extremity literature, DRIL is considered the most effective surgical approach when preservation of access is essential, with more durable results as compared with other strategies. The procedure reestablishes distal perfusion while maintaining access flow, yielding high rates of symptom resolution and long-term access preservation (>80%).^26^^,^^27^ This result contrasts with other options, including banding procedures or proximalization of inflow, which may require more reinterventions.^28^

With modern advances in HD access management, DRIL has become less commonly performed in upper extremity cases and is exceedingly rare in the lower extremity.^29^ Fewer than 10 cases of femoral DRIL have been reported in the literature. In this patient, the technique was adapted using an external iliac-to-distal SFA bypass with interval ligation of the mid-SFA distal to the AVG inflow. This modification achieved rapid symptom relief and sustained patency, supporting DRIL as a feasible and effective strategy for femoral DASS when performed with careful anatomical planning.^16^

## Conclusions

Lower extremity steal syndrome is a rare but serious complication of femoral AVGs. This case demonstrates the successful adaptation of the DRIL procedure at the femoral level, providing limb salvage and preservation of HD access in a patient with exhausted upper extremity options. The durable outcome in this patient adds to limited existing literature supporting femoral DRIL as a viable option for lower extremity DASS. Further studies exploring surgical technique and outcomes would be required to define long-term patency and outcomes in larger cohorts.

## Funding

None.

## Disclosures

None.
